# Huge hydro pneumoperitoneum reported as complication of perforated duodenal ulcer with interesting images

**DOI:** 10.11604/pamj.2019.34.217.20829

**Published:** 2019-12-30

**Authors:** Islam Hussam Elrobaa, Abdulhadi Hayder Khan, Muayad Kassim Ahmad, Abraham John Elamatha, Mohamed Darwish Elserhy, Elfatih Eltahir, Khalid Abdulnour Saifeldeen

**Affiliations:** 1 Al Wakra Hospital, Hamad Medical Corporation, Al Wakra, Qatar

**Keywords:** Abdominal pain, perforated duodenal ulcer, hydro pneumoperitoneum

## Abstract

This case has interesting images of huge hydro pneumoperitoneum. It is a rare view in the medical practice and a good point to learn one of the complications of perforated duodenal ulcer and failure of omental patch operation. We reported a case of acute abdominal pain with hydro pneumoperitoneum that appeared as an air fluid line in X-ray. The patient had an omental patch surgery. Two days after the operation he got severe abdominal pain. The X-ray images showed significant huge hydro pneumoperitoneum. He underwent urgent surgical intervention for exploration that detected a large amount of gases, a biliary free fluid and a leak from duodenal ulcer. Omental buttressing was then performed.

## Introduction

A hydro pneumoperitoneum refers to free air and fluid in the peritoneal cavity [1]. Whereas pneumoperitoneum refers to air in peritoneal cavity [1]. Both hydro pneumoperitoneum and pneumoperitoneum indicate perforated viscus. It is an important sign that helps to detect the pathological cause of acute abdominal pain. The patient who has this sign may need urgent surgical intervention or if not the patient may die [2]. Usually we can see this sign in chest X-ray as air under diaphragm and it is the famous sign for pneumoperitoneum [3]. The air fluid line in the chest-abdomen X-ray refers to hydro pneumoperitoneum which is less common in perforated duodenal ulcer than pneumoperitoneum [4]. We report a case of acute abdominal pain with hydro pneumoperitoneum that appeared as air fluid line in X-ray. The patient had omental patch operation. Two days after the operation he got severe abdominal pain. The X-ray and CT scan images showed significant huge hydro pneumoperitoneum. He underwent urgent surgical intervention for exploration that detected a large amount of gases, a biliary free fluid and a leak from duodenal ulcer. Omental buttressing was then performed.

## Patient and observation

**Figure 1:**
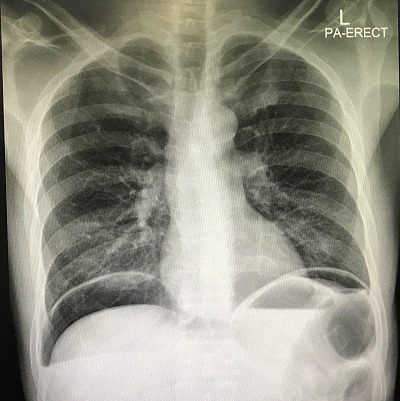
First X-ray was in ED which showed pneumoperitoneum with air fluid line

**Figure 2:**
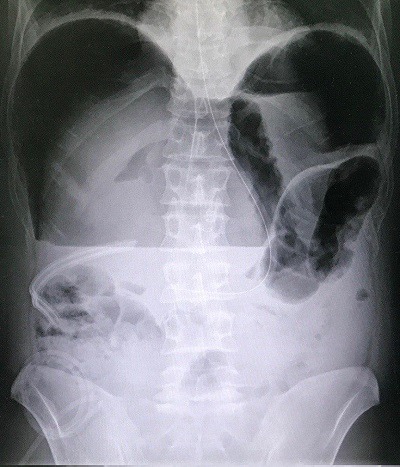
Second X-ray was 2 days after the first operation i.e. omental Graham patch operation, which showed a significant huge hydro pneumoperitoneum

**Figure 3:**
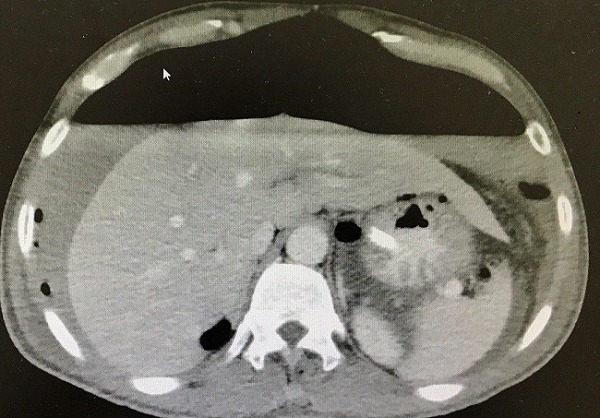
CT Scan was 2 days after omental Graham patch operation, showing massive hydro pneumoperitoneum

**Figure 4:**
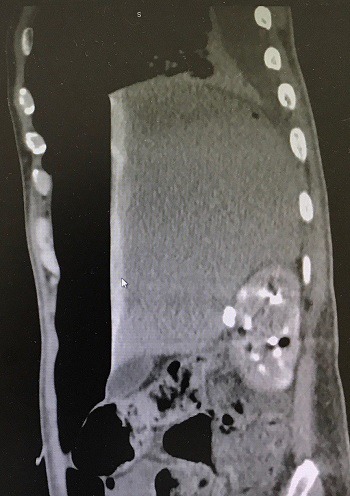
CT Scan was 2 days after the first operation, omental Graham patch operation, showing massive hydro pneumoperitoneum

**Figure 5:**
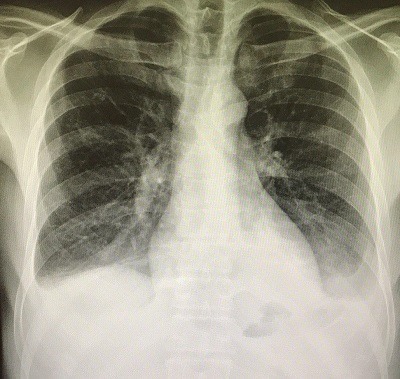
Third X-ray was 6 days after the second operation, omental buttressing, that showing no air or fluid in abdominal cavity

A 47-year-old man presented to the emergency department with acute abdominal pain and vomiting. On examination, vital signs were stable, chest was clear and the abdomen was rigid and there was tenderness. Focused bed side ultrasound showed large amount free fluid. Chest X-ray showed an average amount of air under diaphragm with air fluid line (Figure 1). The patient denied any trauma. He was sent to theater for operation. Upon surgery, free fluid with pus and perforated duodenal ulcer was found. Omental Graham patch was done to repair the perforated ulcer. Patient was better after the operation and he passed flatulence. Two days after the operation he passed stool and then suddenly he got severe abdominal pain. He had a distention abdomen with significant tympanic sound by percussion. X-ray (Figure 2) and computed scan (CT) scan showed a huge amount of hydro pneumoperitoneum (Figure 3, Figure 4). He underwent another surgery for exploration which showed a large pneumoperitoneum, a large amount of biliary free fluid and leak from duodenal ulcer at superior portion of previous repair. Omental buttressing was done and the patient was OK after that (Figure 5).

## Discussion

Pneumoperitoneum is one of complications of perforated duodenal ulcer [5]. The air under diaphragm is the famous sign of pneumoperitoneum and sometime could be missed or can't be seen in X-ray the air is a small amount of which in that case, a CT scan is the diagnostic of choice [6]. Usually, a perforated duodenal ulcer is presented by pneumoperitoneum. Sometimes, it may be presented by hydro pneumoperitoneum which appear as air fluid line in the images [7]. A perforated duodenal ulcer is the most common cause of pneumoperitoneum. Other causes are bowel obstruction, ruptured diverticulum, penetrating trauma, ruptured inflammatory bowel disease (mega colon), necrotizing enterocolitis, bowel cancer, ischemic bowel, after laparotomy, after laparoscopy, break down of surgical anastomosis, bowel injury after endoscopy, peritoneal dialysis, vaginal insufflation, peritoneal infection, from chest (e.g. bronchopleural fistula and noninvasive positive air pressure) etc. [8].

This case has presented hydro pneumoperitoneum as a complication of perforated duodenal ulcer. The bed side ultrasound showed large amount of free fluid in the whole abdomen. The emergency physician had suspicion of abdominal trauma which the patient denied. Even after the omental Graham patch the ulcer had recurrence with significant huge hydro pneumoperitoneum. The omental buttressing was done. Patient was OK after that. Regarding the evidence-based practice, omental patch repair does not correct the underlying process that causes perforation, and ulcers may recur. In a study of 94 patients with perforated foregut ulcers (53 gastric and 41 duodenal), of whom 77 [82%] were treated by omental patching alone, Smith *et al.* documented a 12% rate of ulcer recurrence after omental patching and a 23% incidence of recurrent symptoms within 44 months [9].

## Conclusion

We have reported the case of a significant huge hydro pneumoperitoneum which is an unusual presentation in perforated duodenal ulcer. Omental patch repair does not correct the underlying process that causes perforation, and ulcers may recur. Emergency physicians and surgeons should be aware of unusual presentation of duodenal ulcer and other diseases. Omental patch operation could induce huge hydro pneumoperitoneum if it fails and the patient had a presentation of hydro pneumoperitoneum.

## Competing interests

The authors declare no competing interests.
